# Schistosome AMPK Is Required for Larval Viability and Regulates Glycogen Metabolism in Adult Parasites

**DOI:** 10.3389/fmicb.2021.726465

**Published:** 2021-09-01

**Authors:** Kasandra S. Hunter, André Miller, Margaret Mentink-Kane, Stephen J. Davies

**Affiliations:** ^1^Department of Microbiology and Immunology, F. Edward Hébert School of Medicine, Uniformed Services University, Bethesda, MD, United States; ^2^Schistosomiasis Resource Center, Biomedical Research Institute, Rockville, MD, United States

**Keywords:** *Schistosoma mansoni*, schistosome, 5′ AMP-activated protein kinase, AMPK (5′-AMP activated kinase), energy metabolism, glycolysis, glycogen, RNA interference

## Abstract

On entering the mammalian host, schistosomes transition from a freshwater environment where resources are scarce, to an environment where there is an unlimited supply of glucose, their preferred energy substrate. Adult schistosome glycolytic activity consumes almost five times the parasite’s dry weight in glucose per day to meet the parasite’s energy demands, and the schistosome glycolytic enzymes and mechanisms for glucose uptake that sustain this metabolic activity have previously been identified. However, little is known of the parasite processes that regulate schistosome glucose metabolism. We previously described the *Schistosoma mansoni* ortholog of 5′ AMP-Activated Protein Kinase (AMPK), which is a central regulator of energy metabolism in eukaryotes, and characterized the developmental regulation of its expression and activity in *S. mansoni*. Here we sought to explore the function of AMPK in schistosomes and test whether it regulates parasite glycolysis. Adult schistosomes mounted a compensatory response to chemical inhibition of AMPK α, resulting in increased AMPK α protein abundance and activity. RNAi inhibition of AMPK α expression, however, suggests that AMPK α is not required for adult schistosome viability *in vitro*. Larval schistosomula, on the other hand, are sensitive to chemical AMPK α inhibition, and this correlates with inactivity of the AMPK α gene in this life cycle stage that precludes a compensatory response to AMPK inhibition. While our data indicate that AMPK is not essential in adult schistosomes, our results suggest that AMPK regulates adult worm glycogen stores, influencing both glycogen utilization and synthesis. AMPK may therefore play a role in the ability of adult schistosomes to survive *in vivo* stressors such as transient glucose deprivation and oxidative stress. These findings suggest that AMPK warrants further investigation as a potential drug target, especially for interventions aimed at preventing establishment of a schistosome infection.

## Introduction

Schistosomiasis is a chronic inflammatory disease caused by infection with parasitic platyhelminths of the genus *Schistosoma.* The species *Schistosoma japonicum, Schistosoma haematobium*, and *S. mansoni* are of primary concern in human medicine as they cause the majority of human schistosomiasis (King, 2011). It is estimated that over 250 million individuals are infected with schistosomes (Colley et al., 2014; Hotez et al., 2014; World Health Organization., 2015), resulting in the loss of over 10 million disability-adjusted life years (DALYs). Infection is characterized by severe inflammation of critical organs, ultimately resulting in the accumulation of irreparable and life-threatening tissue damage if left untreated (King, 2015).

Humans contract schistosomiasis via contact with fresh water environments where the aquatic infectious larval stages known as cercariae are found. Cercariae penetrate intact human skin (McKerrow and Salter, 2002) and, once inside the body, the juvenile schistosomes migrate through the vasculature for several weeks until the vessels that are their preferred final destination are reached. Once mature, adult male and female schistosomes pair and female worms begin to produce hundreds to thousands of eggs per day. Some eggs successfully exit the human body to continue the schistosome life cycle in the outside environment (King, 2011). Many eggs, however, are unable to exit the host and become trapped in host tissues where they incite immune responses, leading to inflammation and granuloma formation. Over time, granulomatous tissue surrounding schistosome eggs becomes fibrotic, resulting in tissue scarring and complications such as hepatosplenomegaly, periportal fibrosis, portal hypertension, and esophageal varices (Colley et al., 2014). Currently, there is only one therapeutic, praziquantel, available to treat schistosomiasis, and this drug is only effective against adult schistosomes. There is no prophylactic available to prevent infection (Trainor-Moss and Mutapi, 2016).

Schistosomes are obligate parasites that rely on their hosts to supply essential factors they are unable to manufacture for themselves. One of the most important nutrients that schistosomes must acquire from the surrounding host environment is glucose, as it is the parasite’s principle energy substrate (You et al., 2014). Previous studies have demonstrated that when glucose access is interrupted, schistosome survival is impaired (Krautz-Peterson et al., 2010). Adult schistosomes consume as much as 20% of their dry weight in glucose per hour, the vast majority of which is funneled into glycolysis (Bueding, 1950). Schistosomes depend almost exclusively on glycolysis to meet the energetic demands of parasite growth and reproduction (Tielens, 1994; You et al., 2014). Previous efforts to identify the energy sources that schistosomes exploit to meet their high metabolic needs revealed that these parasites are largely unable to metabolize lipids, leaving glucose metabolism as their sole mechanism of energy production (Bexkens et al., 2018).

Because of the importance of glucose metabolism in schistosomes, previous research has focused on elucidating the mechanisms by which schistosomes acquire glucose, as well as factors that might influence glucose uptake, in order to uncover potential opportunities for intervention. Previous studies have identified schistosome insulin receptors (Ahier et al., 2008; You et al., 2010), parasite glucose transporters that facilitate glucose uptake (Skelly et al., 1994; Skelly et al., 1998), and schistosome glycolytic enzymes (You et al., 2014). However, little is known about the regulatory mechanisms that control schistosome energy metabolism. Analysis of schistosome genomic sequences have predicted that the regulatory networks that control energy metabolism in other eukaryotes are conserved in schistosomes (Berriman et al., 2009; The Schistosoma japonicum Genome Sequencing Functional Analysis Consortium, 2009; Young et al., 2012), but little has been done to characterize these networks in schistosomes or test their potential as targets for novel interventions. Targeting the regulation of intracellular energy metabolism, and specifically glucose metabolism, is useful for the treatment of diabetes and other related metabolic diseases (Zhang et al., 2009; Hardie, 2013; Coughlan et al., 2014). A similar approach is being explored for the treatment of various types of cancer (Hamanaka and Chandel, 2012; Li et al., 2015; Yung et al., 2016). Given these metabolic regulatory mechanisms are conserved in eukaryotes, this approach could also be useful for targeting schistosomes, as well as other eukaryotic pathogens.

In mammals and other eukaryotes, numerous pathways are involved in the regulation of glucose uptake and metabolism, including the insulin signaling pathway (Saltiel and Kahn, 2001), protein kinase A (PKA) (Jiang and Zhang, 2003; Kunkel et al., 2019), Akt (Schultze et al., 2012; Swiderska et al., 2018), and 5′ AMP-activated Protein Kinase (AMPK) (Hardie, 2011; Kishton et al., 2016). AMPK in particular is conserved in all eukaryotes, and is the intracellular master regulator of cellular energy metabolism and energy homeostasis (Hardie, 2014; Herzig and Shaw, 2018). AMPK is a heterotrimeric protein, comprised of α, β, and γ subunits that each perform a unique function in regulating the cellular energy balance. The γ subunit senses the cellular energy status via direct binding of ATP, ADP and AMP. In instances where cellular ATP levels are low, AMP and ADP out compete ATP for binding to the AMPK γ subunit, leading to phosphorylation and activation of the catalytic α subunit kinase domain (Hardie, 2011; Hardie et al., 2012; Gowans and Hardie, 2014). Activation of AMPK kinase activity leads to multiple metabolic outcomes that achieve the common goal of dampening anabolic processes to decrease ATP consumption (e.g., gluconeogenesis, lipid synthesis, and glycogen synthesis), while simultaneously stimulating catabolic processes to promote ATP generation (e.g., glycolysis, fatty acid oxidation, and glycogen breakdown) (Lage et al., 2008). Mammalian AMPK activation results in increased glucose uptake, indirect stimulation of the key glycolytic enzyme phosphofructokinase-1, inhibition of glycogen synthesis via phosphorylation of glycogen synthase, and activation of glycogen breakdown via phosphorylation of glycogen phosphorylase (Jeon, 2016). While these effects are mainly mediated by the kinase activity of the AMPK α subunit, the β subunit also plays a significant role in regulating glycolysis by interacting with cellular glycogen reserves. A glycogen binding domain in the AMPK β subunit allows for the direct association of AMPK with glycogen, allowing AMPK to gauge cellular glycogen stores (Polekhina et al., 2003; McBride and Hardie, 2009; McBride et al., 2009; Janzen et al., 2018).

Previously, we reported the presence of an AMPK ortholog in the human parasite *S. mansoni* (Hunter and Davies, 2018). While the schistosome AMPK α subunit exhibits high levels of identity and similarity to the AMPK α of humans and other species, the schistosome sequence diverges considerably in the C-terminal half of the protein due to the presence of approximately 100 additional, non-conserved amino acids. Furthermore, we showed that while the *S. mansoni* AMPK α protein is present in all developmental stages of the parasite, the gene is not actively expressed in the infectious cercaria and early intra-mammalian stages. Lastly, we demonstrated that schistosome AMPK activity is vulnerable to modulation by host factors and chemical agents (Hunter and Davies, 2018).

Because AMPK is a central regulator of energy metabolism in other eukaryotes, we hypothesized that schistosome AMPK is critical for the regulation of parasite glycolysis and could therefore serve as a novel therapeutic target for disrupting schistosome energy metabolism. To test this hypothesis, we sought to characterize the relationship between schistosome AMPK activity and parasite glycolysis, and ascertain the metabolic and phenotypic consequences of targeting parasite AMPK activity. Our data suggest that the schistosome AMPK fulfills different roles in larval and adult parasites, being required for viability in the former and regulating cellular glycogen stores in the latter.

## Materials and Methods

### Ethics Statement

All animal procedures were performed in accordance with guidelines outlined in the National Research Council’s *Guide for the Care and Use of Laboratory Animals* (The National Academies Press) and were pre-approved (as protocol number MIC-17-504) by the Institutional Animal Care and Use Committee (IACUC) of Uniformed Services University (permit number A3448-01). Animals were euthanized by intraperitoneal injection of sodium pentobarbital euthanasia solution, followed by cervical dislocation or bilateral thoracotomy, in accordance with the American Veterinary Medical Association Guidelines for the Euthanasia of Animals.

### Parasite Materials

Albino *Biomphalaria glabrata* infected with *S. mansoni* miracidia (NMRI strain) were provided by Biomedical Research Institute (BRI; Rockville, MD, United States) within a week post-miracidial exposure. At 6 weeks post-infection, *B. glabrata* snails were exposed to light for 1–1.5 h to promote the emergence of cercariae. Cercariae were collected in a conical tube and either used to infect mice or were transformed into schistosomula. Mice were infected with *S. mansoni* via tail skin exposure to 135 cercariae for 30 min, after which they were returned to their cages. After 8 weeks, adult schistosomes were recovered from infected mice via portal vein perfusion with RPMI medium. Cercariae were mechanically transformed into schistosomula via 20–30 passages through an emulsification needle using 30 ml syringes (Luer-Lok Tip, BD). Cercarial heads (newly transformed schistosomula) were separated from tails via multiple rounds of swirling in RPMI + 1% HEPES buffer + 2% antibiotic/antimycotic [Antibiotic Antimycotic Solution (100×), Stabilized, containing 10,000 units penicillin, 10 mg streptomycin and 25 μg amphotericin B per ml, Invitrogen] using deep petri dishes.

### Chemical Compounds

Metformin hydrochloride (Tocris Bioscience, catalog no. 2864) was dissolved in ultrapure distilled water (Invitrogen) to a stock concentration of 100 mM and stored at −20°C. 2-Deoxy-D-glucose (2-DG) (Sigma Aldrich, catalog no. D8374) was dissolved in ultrapure distilled water (Invitrogen) to a stock concentration of 500 mM and stored at −20°C. Stock solutions of 2-DG were discarded after one freeze-thaw cycle. 5-Aminoimidazole-4-carboxamide ribonucleotide (AICAR) (Sigma Aldrich, catalog no. A9978) was solubilized in ultrapure distilled water (Invitrogen) to a stock concentration of 33 mM and stored at −20°C. Dorsomorphin was supplied in either dimethyl sulfoxide (DMSO) -soluble (Abcam, catalog no. ab120843) or water-soluble forms (dorsomorphin dihydrochloride, Tocris Bioscience, catalog no. 3093). Either DMSO (Fisher Scientific) or ultrapure distilled water (Invitrogen) were used as appropriate to produce stock solutions of 2.5 mM. Aliquots of DMSO-soluble dorsomorphin were stored at −20°C. Dorsomorphin dihydrochloride stock solutions were prepared fresh when possible or stored at −80°C and thawed after one freeze-thaw cycle. Glycogen Phosphorylase Inhibitor (2-chloro-4,5-difluoro-N-[[[2-methoxy-5-[[(methylamino)carb onyl]amino]phenyl]amino]carbonyl]-benzamide) (EMD Millipore Corp, catalog no. 361515) was dissolved in DMSO to make a 2.4 mM stock solution that was stored at 4°C.

### Agilent Seahorse XFe96 Glycolysis Assay

An Agilent Seahorse XFe96 Analyzer was used to assess the metabolic output of adult and juvenile schistosomes in response to AMPK modulating factors. To determine the rate of glycolysis, the extracellular acidification rate was measured over time using the Agilent Seahorse XFe96 Extracellular Flux Assay Kit cartridge (Agilent Technologies, catalog no. 102905-100) and Agilent Seahorse XFe96 Spheroid Microplates (Agilent Technologies, catalog no. 102978-100). Twenty-four hours prior to the start of an experiment, cartridge probes were soaked in 200 μl Ultrapure Distilled Water (Invitrogen) at 37°C. The water was replaced with 200 μl Agilent Seahorse XF Calibrant (Agilent Technologies, catalog no. 100840-000) an hour prior to starting the experiment. Adult schistosomes were recovered by perfusion from infected mice immediately prior to the start of the experiment and washed with 1 × PBS to remove perfusion fluid. Either a single schistosome pair, containing one male and one female adult schistosome, or 500 newly transformed schistosomula were placed in each well of an Agilent Seahorse XFe96 Spheroid Microplate with 180 μl of Seahorse XF DMEM medium (Agilent Technologies, catalog no. 103575-100) supplemented with 1 mM Seahorse XF pyruvate solution (Agilent Technologies, catalog no. 103578-100), 10 mM Seahorse XF glucose solution (Agilent Technologies, catalog no. 103577-100), 2 mM Seahorse XF L-glutamine solution (Agilent Technologies, catalog no. 103579-100), and 1× antibiotic/antimycotic. Cartridge compound-administration chambers were loaded with 25 μl of either media or AMPK modulating compounds at pre-calculated concentrations to achieve desired final concentrations in a final volume of 205 μl of media before beginning Seahorse XFe96 Analyzer experiment.

### Agilent Fluorescent Glycolysis Assay

Glycolytic rates, both basal and post-exposure to AMPK-modulating compounds, of adult schistosomes and newly transformed schistosomula were measured using the pH-Xtra Glycolysis Assay (Agilent Technologies, catalog no. PH-200-4). Manufacturer’s instructions described in the pH Xtra Glycolysis User Manual were modified to adapt the standard protocol to use with parasites. Briefly, either 2 adult worm pairs or 1,000 schistosomula were distributed per well in a black, clear bottom 96-well plate (Corning). PH-Xtra Reagent was solubilized in 1 ml ultapure distilled water (Invitrogen). One pH-Xtra respiration buffer tablet was fully dissolved in 50 ml Ultrapure Distilled Water (Invitrogen) and warmed to 37°C. The pH was adjusted to 7.4 using KOH and NaOH. After pH-adjustment, the respiration buffer solution was sterilized via filtration through a 0.22 μm filter. Parasites were washed twice in warmed respiration buffer and resuspended in 60 μl buffer + 10 μl pH-Xtra reagent per well, to maximize fluorescent signal. Compounds were added to respective wells at volumes calculated to achieve desired final concentrations. Plates were kept at 37°C and fluorescence was measured every 15 min using a Spectramax M2 spectrophotometer (Molecular Devices) at an excitation/emission wavelength of 350/615 nm.

### *S. mansoni* AMPK Kinase Activity Assay

The AMPK was immunoprecipitated from adult *S. mansoni* lysate using anti-AMPK α antibodies according to the immunoprecipitation protocol we described previously (Hunter and Davies, 2018). Briefly, 1 μg anti-AMPK α (Invitrogen, catalog no. PA5-36045) was incubated with 50 μg of pre-cleared adult schistosome lysate prepared in 1% NP-40 lysis buffer for 1 h at 4°C. The lysate/antibody was then incubated with a 50% nProtein A Sepharose Fast Flow (GE Healthcare) bead slurry for 1 h at 4°C with gentle mixing to precipitate immune complexes. After incubation, the mixture was centrifuged at 12,000 × *g* for 30 s to isolate the bead-bound *S. mansoni* AMPK α-antibody complexes. After washing five times in 1% NP-40 buffer, bead-bound immune complexes were resuspended in 1× kinase buffer (prepared from 10× kinase buffer, Cell Signaling Technologies, catalog no. 9802S) supplemented with adenosine triphosphate (ATP) (Sigma Aldrich, catalog no. A1852) and adenosine monophosphate (AMP) (Sigma Aldrich, cat no. 01930, each to a final concentration of 200 μM). To determine the effects of dorsomorphin on AMPK activity, dorsomorphin was added to the kinase buffer/immune complex mixture to a final concentration of 10 μM. Following the addition of 0.2 μg acetyl-CoA carboxylase (ACC; Sigma-Aldrich, catalog no. A6986) as substrate, the mixture was incubated at 37°C for 10 min, after which the reaction was terminated by incubation in 1× lithium dodecyl sulfate (LDS) sample buffer (25% 4× LDS sample buffer, 65% ddH_2_O, 10% beta-mercaptoethanol) at 95°C for 5 min. The supernatant was separated from nProtein A Sepharose beads by centrifugation at 12,000 × *g* for 30 s, transferred to a new tube, and analyzed via Western blot to assess ACC phosphorylation status. The kinase assay outlined above was adapted from a protocol previously described by Hardie et al. (2000).

### Preparation of Parasite Protein Extracts

Immediately post-perfusion, adult schistosomes were either directly used to extract protein or flash frozen in liquid nitrogen or stored at −80°C for protein preparation at a later date. Whole protein extracts were prepared by homogenizing parasites in 1× LDS sample buffer (25% 4× LDS sample buffer, 65% ddH_2_O, 10% beta-mercaptoethanol), supplemented with 100× Halt protease inhibitor cocktail (Invitrogen) and 100× phosphatase inhibitor to a final concentration of 1×, using an electric homogenizer. To further homogenize remaining particulate matter, samples were briefly subjected to one round of sonication at intervals of 10 s for 4 min. Samples were incubated for 10 min at 95°C. Insoluble material was removed from the lysate via centrifugation for 5 min at 1,800 × *g*. Schistosomula protein was similarly prepared, however, samples were homogenized in boiling 1× LDS sample buffer to reduce the activity of cercarial proteases.

### Western Blotting

Protein concentrations of parasite lysates were determined using the Pierce 660 nm Protein Assay Kit (Thermo Scientific). Protein separation by SDS-PAGE was performed by loading 5–10 μg of sample into 10% Bis-Tris gels (NuPAGE) in 1× MES Buffer (Novex) and run at 200 V for 1 h. Immediately following separation, proteins were transferred onto Invitrolon polyvinyl difluoride membranes (PVDF) (Novex) for approximately 90 min at 25 V, 160 mA. After transfer, PVDF membranes were blocked in Startingblock TBS Blocking Buffer (Thermo Scientific) with 0.05% Tween-20 for 30 min. Blocked membranes were probed for 1 h or overnight with either AMPK alpha- 1,2 rabbit polyclonal antibody (Invitrogen, catalog no. PA5-36045) diluted 1:500, phospho-AMPK alpha (T172) rabbit monoclonal antibody (Cell Signaling Technology, catalog no. 2535) diluted 1:1000, Acetyl CoA carboxylase rabbit monoclonal antibody (Cell Signaling Technology, catalog no. 3676) diluted 1:1000, phospho-Acetyl CoA (Ser179) rabbit monoclonal antibody (Cell Signaling Technology, catalog no. 11818) diluted 1:1000, or beta tubulin rabbit polyclonal antibody (Abcam, catalog no. ab15568) diluted 1:5000. After primary antibody incubation, membranes were washed five times in 1× TBST Buffer (Thermo Scientific) then probed with Goat anti-rabbit IgG (H + L) cross-adsorbed HRP secondary antibody (Thermo Scientific, catalog no. G21234) diluted 1:2000 for 1 h. All antibodies were diluted in Startingblock TBS Blocking Buffer with 0.05% Tween-20. Finally, membranes were incubated in SuperSignal West Pico PLUS Chemiluminescent Substrate (Thermo Scientific) for 5 min to detect bound antibody. Membranes were imaged using an Amersham Imager 680 blot and gel imager (GE Healthcare Life Sciences). Densitometry measurements were calculated using ImageJ Software (Schneider et al., 2012).

### *In vitro* Dorsomorphin Parasite Treatment

Adult schistosomes were transferred to 12-well tissue culture treated plates immediately post-perfusion in 2 ml DMEM supplemented with 10% heat-inactivated FBS and 1% antibiotic/antimycotic. Newly transformed schistosomula were placed in 12- or 24-well tissue culture treated plates in 2 ml Modified Basch Medium (Basch, 1981) immediately following transformation. Dorsomorphin was added directly to media in various concentrations. Plates were swirled to evenly distribute dorsomorphin throughout media and incubated at 37°C, 5% CO_2_. Schistosomes were collected at 2 and 24 h post-exposure to dorsomorphin and either phenotypically scored or flash frozen in liquid nitrogen. Frozen parasites were stored at −80°C for later use.

### Parasite RNA Extraction

Total RNA was extracted from frozen parasite samples according to the manufacturer’s technical bulletin (Sigma Aldrich). Briefly, two or more adult schistosome pairs per sample were homogenized in 1 ml RNAzol RT (Sigma Aldrich, catalog no. R4533) using an electric homogenizer. The resulting homogenate was mixed vigorously with 400 μl water for 15 s then stored at room temperature for 15 min before centrifugation at 12,000 × *g* for 15 min. The supernatant was transferred to a new tube and mixed with 1 mL isopropanol to precipitate RNA. After incubation for 10 min at room temperature followed by centrifugation at 12,000 × *g* for 10 min, the RNA pellet was washed three times with 75% ethanol. After the final wash, the RNA pellet was dissolved in ultapure distilled water (Invitrogen). To further purify and concentrate extracted RNA, the RNeasy MinElute Cleanup Kit (Qiagen) was used according to the supplied “RNA Cleanup” protocol. The optional DNase digestion step was performed prior to starting the “RNA Cleanup” protocol using the RNase-Free DNase Set (Qiagen) according to the supplied protocol. The resulting RNA was stored at −80°C for later use.

### RT-PCR Quantification of AMPK α Transcripts

Quantification of AMPK α transcripts was performed using RT-PCR according to our previously described protocol (Hunter and Davies, 2018). Briefly, 500 ng parasite RNA was used as template for cDNA synthesis reactions using the High Capacity RNA-to-cDNA Kit (Applied Biosystems). PCR reaction was performed using TaqMan Fast Universal PCR Master Mix (Thermo Fisher Scientific) with 1 μl cDNA as template and primers targeting nucleotide positions 751–815 of *S. mansoni* AMPK α (forward 5′-AGAATGATTACTGTGGACCCGATT-3′ and reverse 5′-AACCACGGATGTCGTCTGATTT-3′) to produce a 64 bp fragment. To detect amplification of the fragment, a TaqMan MGB probe conjugated to an oligo sequence positioned at nucleotide 776 (5′-AACGTGCAACCATAGAA-3′) was used. AMPK α copy numbers were calculated using a standard curve. All reactions were performed using a 7500 Fast Real-Time PCR System (Applied Biosystems). Subsequent quantification of transcripts was performed using GraphPad Prism software, version 7.05 (GraphPad Software, Inc).

### Parasite Phenotypic Scoring

Phenotypic screening of parasites following dorsomorphin exposure and RNAi was performed according to a protocol adapted from Kyere-Davies et al. (2018). Briefly, single word descriptors for parasite phenotypic characteristics such as movement, shape, translucence, and orientation were used to characterize treatment effects that could be observed visually. For each characteristic, parasites were assessed and assigned a number ranging from 1 = unaffected/healthy to 4 = severely affected or damaged. The average was calculated in order to determine an overall severity score. Parasites were assessed using the described phenotypic scoring system at 2 and 24 h post *in vitro* exposure to dorsomorphin. Following RNAi, the phenotypic scoring system was used to assess the impact of AMPKα transcript reduction at 24, 48, and 72 h post-RNAi. For example, an adult schistosome pair on its side and exhibiting uncoordinated movement would be assigned a higher severity score than a pair that remained suctioned to the tissue culture plate, the typical orientation of a healthy schistosome pair.

### Preparation of DsiRNA for RNAi

Dicer-substrate short interfering RNA (DsiRNA) oligos targeting the *S. mansoni* AMPK α sequence (GenBank accession number MH445971) at three different sequence positions, 26 nucleotides in length, were prepared by Integrated DNA Technologies (IDT): DsiRNA #1- CD.Ri.195363.13.5 (forward 5′- rGrUrCrArArArGrUrUrGrGrArArUrUrCrArCrArArArUrCTA -3′ and reverse 3′-rUrArGrArUrUrUrGrUrGrArArUrUrCrCrAr ArCrUrUrUrGrArCrUrU-5′) targeting nucleotide positions 120–145, DsiRNA #2- CD.Ri.195363.13.4 (forward 5′-rCrArCr UrGrGrArUrCrUrGrCrUrArGrUrCrCrArArCrCrAAT-3′ and reverse 3′-rArUrUrGrGrUrUrGrGrArCrUrArGrCrArGrArUrC rCrArGrUrGrCrU-5′) targeting nucleotide positions 1805–1830, DsiRNA #3- CD.Ri.195363.13.2 (forward 5′-rArGrUrArUrUrUr ArArArGrCrArArUrGrArArUrUrCrArCTT-3′ and reverse 3′-r ArArGrUrGrArArUrUrCrArUrUrGrCrUrUrUrArArArUrArCr UrUrC-5′) targeting nucleotide positions 1,499–1,524. Scrambled Negative Control DsiRNAs were also supplied by IDT. DsiRNAs were reconstituted in Duplex Buffer (Integrated DNA Technologies) to a final concentration of 100 μM then heated at 95°C for 2–3 min and allowed to cool at room temperature for 1 h before use.

### Adult Schistosome RNAi

Immediately post-perfusion, freshly isolated adult schistosomes were collected and separated into microcentrifuge tubes, 5–7 pairs per tube. Parasites were washed three times with Opti-MEM to ensure serum removal. After washing, parasites were transferred to 4 mm electrode gap cuvettes and resuspended in 100 μl Opti-MEM supplemented with 6 μl of 100 μM DsiRNA stock solution per sample. To test the effect of all three oligos combined, 2 μl of each oligo’s 100 μM stock were used. Parasites in Opti-MEM were gently mixed to evenly distribute DsiRNA, then electroporated at room temperature with a single square wave pulse for 20 ms at 125 V using the Gene Pulser Xcell Electroporation System (Bio-Rad). Parasites were immediately removed from the cuvette, transferred to a 12 well tissue-culture treated plate in 2 ml complete DMEM medium, and incubated at 37°C, 5% CO_2_. At 24, 48, and 72 h post-electroporation, parasites were collected to assess effects of RNAi on AMPK α transcript abundance. At 7 days post-electroporation, parasites were collected to assess AMPK α protein levels or flash frozen in liquid nitrogen and stored at −80°C for later use.

### Measuring Parasite Glycogen Content

Adult schistosomes in culture or newly transformed schistosomula were collected in microcentrifuge tubes. Parasites were washed twice with 1× PBS to remove trace amounts of media, then flash frozen in liquid nitrogen to prevent breakdown of glycogen. Frozen parasites were resuspended in 100 μl ultrapure distilled water (Invitrogen) and homogenized using an electric homogenizer. Upon complete homogenization of parasite tissue, samples were boiled for 5 min then centrifuged at 4°C at 13,000 × *g* for 5 min to remove insoluble material. Glycogen concentration of supernatants were determined using the Sigma-Aldrich Glycogen Assay Kit (catalog no. MAK016) according to the manufacturer’s instructions for colorimetric readout. The colorimetric signal produced, which is proportional to the glycogen present, was read at 570 nm using a Spectramax M2 spectrophotometer (Molecular Devices). For normalization purposes, sample protein concentrations were determined using the Pierce BCA Protein Assay (Thermo Scientific).

### Cloning and Sequencing of *S. mansoni* AMPK β

Cloning, sequencing, and analysis of *S. mansoni* AMPK β were performed using the same approach previously described for AMPK α (Hunter and Davies, 2018). Briefly, adult *S. mansoni* cDNA was used as the template for amplifying the full-length AMPK β cDNA sequence. To prepare cDNA, parasite RNA was extracted from adult schistosomes as previously detailed. Crude RNA extracts were further purified using the RNeasy MinElute Cleanup Kit with DNase I digestion (Qiagen). 1 μg RNA was used as the template for cDNA synthesis using the High-Capacity cDNA Reverse Transcription Kit (Applied Biosystems). Using the putative *S. mansoni* AMPK β mRNA sequence (Smp_170160) predicted by the Schistosome Genome Consortium (Berriman et al., 2009) and matching expressed sequence tags (ESTs) identified in the NCBI EST database, a consensus sequence was obtained that extended the predicted mRNA by 77 bp at the 5′ end and 43 bp at the 3′ end. This consensus sequence was used as template to design primers that amplified AMPK β in its entirety. Two overlapping fragments, one 573 bp fragment (forward primer: 5′-GGACCCGTGGCTAATTTCCA-3′, targeting the 5′ UTR, and reverse primer: 5′-TTTTGAACACCGGTCGGACT-3′) and one 608 bp fragment (forward primer: 5′-AGTCCGACCGGTGTTCAAAA-3′ and reverse primer: 5′-ACTCTCTCCTCTTCCTCCTCT-3′, targeting the 3′ UTR), were amplified by conventional PCR using Accuprime Pfx Supermix (Thermo Fisher Scientific). Purified fragments were then cloned into pCR-BluntII-TOPO vector and sequenced using a BigDye Terminator cycle sequencing kit (Thermo Fisher) and T7 primers. Sequence data was analyzed and contigs assembled using Geneious software, version 11.0.5 (Biomatters Ltd.). Sequence alignments comparing the AMPK β sequence of *S. mansoni* with those of other organisms were performed using Geneious software.

### Statistical Analysis

For both Agilent Seahorse XFe96 and fluorescent glycolysis assay graphs, area under the curve (AUC) analyses were performed. For Seahorse XFe96 data, the AUC from the time point immediately preceding the addition of drug until experiment’s completion was calculated. For fluorescent assay data, the AUC for the entire experiment beginning at time point “0” until experiment’s completion was calculated. A one-way ANOVA was performed on the calculated AUCs using the untreated group as the reference for comparison. For Western blot data derived from the kinase assay, dorsomorphin treatment, and RNAi experiments, densitometry was performed using ImageJ software (Schneider et al., 2012) to quantify band intensity. Densitometry data for each experimental condition was normalized to one of the control conditions tested, so that data from repeat experiments could be compared. Data from each repeat experiment (at least three independent biological replicates were performed in every case) were considered to be paired repeated measures and were analyzed using paired t tests. For quantitative PCR data, standard curves were used to calculate absolute transcript number in each sample. For each source of RNA, at least three independent biological replicates were tested. The data for each RNA source were first tested for evidence of significant differences in variance, using F tests. Because no significant differences in variance were found, the differences in transcript number between RNA sources were tested using parametric unpaired t tests. For glycogen assay data, three or more biological replicates were tested. The data for each parasite glycogen point were first tested for significant differences in variance, then, tested for significant differences between glycogen content using parametric unpaired t tests. All statistical analyses were performed using GraphPad Prism software, version 7.05 (GraphPad Software, Inc.).

## Results

### Inhibition of Schistosome AMPK Results in Increased Parasite Glycolysis

**FIGURE 1:**
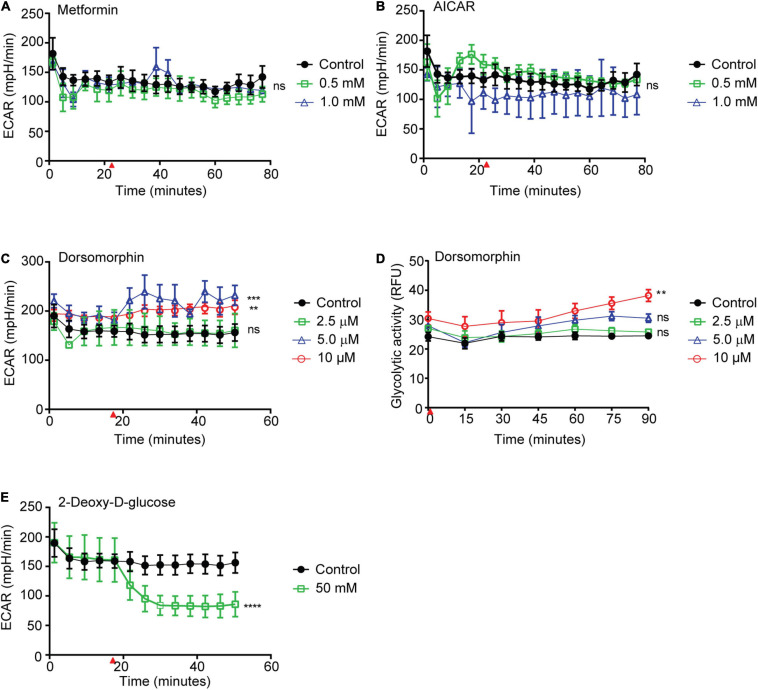
Effects of AMPK agonists and inhibitor on *S. mansoni* glycolytic activity. **(A–C)** The extracellular acidification rate (ECAR) of freshly isolated adult *S. mansoni* pairs exposed to metformin **(A)**, AICAR **(B)**, and dorsomorphin **(C)**, were measured using Agilent Seahorse XFe96 system. **(D)** Extracellular acidification, measured in relative fluorescence units (RFU), of freshly isolated adult schistosome pairs after exposure to dorsomorphin, measured using Agilent pH-Xtra Glycolysis Assay. **(E)** ECAR of freshly isolated adult *S. mansoni* pairs exposed to 2-Deoxy-D-glucose. Red arrows in **(A–C)**, **(E)** indicate time point at which each test compound was introduced into parasite culture media. Individual data points shown are the average of at least 3 biological replicates. Each graph is representative of at least 2–3 independent experiments. ^ns^*P* > 0.05, **P ≤ 0.01, ***P ≤ 0.001, ****P ≤ 0.0001.

Previously, we demonstrated that phosphorylation of *S. mansoni* AMPK α was increased *in vitro* when adult schistosomes were exposed to chemical AMPK agonists or deprived of glucose (Hunter and Davies, 2018). If schistosome AMPK regulates parasite metabolic activity in a manner similar to the AMPK of other organisms, we predicted that increased schistosome AMPK activity would be associated with increased parasite glycolytic activity, while reduced AMPK activity would lead to diminished glycolysis (Hu et al., 2019). To test this hypothesis, we monitored the glycolytic activity of adult schistosomes exposed to the following AMPK modulatory factors: (King, 2011) metformin, a biguanide with antihyperglycemic activity that is thought to directly activate AMPK in mammals; (World Health Organization., 2015) 5-aminoimidazole-4-carboxamide (AICAR), an inosine monophosphate intermediate that stimulates mammalian AMPK by mimicking increased cellular AMP concentrations; and (Colley et al., 2014) dorsomorphin, a pyrazolopyrimidine that potently and reversibly inhibits AMPK by binding directly to the activation site of the α subunit kinase domain (Handa et al., 2011). To monitor schistosome glycolytic activity, we used the Agilent Seahorse platform to detect changes in the extracellular acidification rate (ECAR) of schistosomes exposed to these compounds *in vitro*. While we had previously shown that metformin stimulated schistosome AMPK α phosphorylation (Hunter and Davies, 2018), metformin exposure had no significant effect on schistosome glycolytic activity (Figure 1A). Similarly, AICAR, another AMPK agonist, also had no significant effect on parasite glycolytic activity (Figure 1B). In contrast, higher concentrations of the AMPK inhibitor dorsomorphin stimulated a paradoxical increase in schistosome glycolytic activity (Figure 1C). To corroborate this unexpected result, we repeated the experiment using the Agilent fluorescent glycolysis assay as an alternative methodology of measuring parasite glycolytic activity. Again, dorsomorphin stimulated an increase in parasite glycolysis (Figure 1D). Because these findings were contrary to our expectations, we confirmed that our experimental system was capable of detecting reduced rates of schistosome glycolysis by monitoring the ECAR of schistosomes exposed to 50 mM 2-DG. As expected, 2-DG exposure induced a significant decrease in glycolytic activity (Figure 1E).

### Dorsomorphin Inhibits *S. mansoni* AMPK Kinase Activity

**FIGURE 2:**
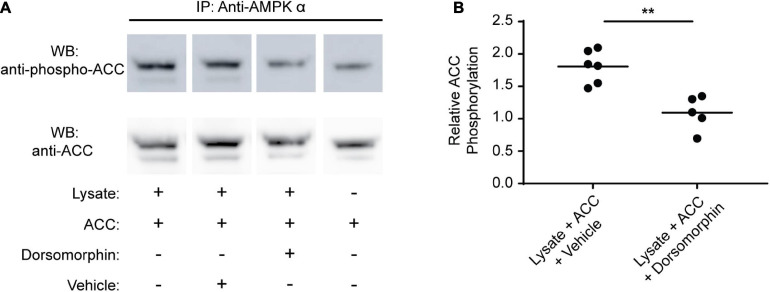
Dorsomorphin inhibits *S. mansoni* AMPK α kinase activity. **(A)** AMPK α was immunoprecipitated from lysates of freshly isolated adult schistosomes using anti-AMPK α, then resuspended in 1× kinase buffer with 0.2 μg acetyl-coA-carboxylase (ACC) in either the presence or absence of 10 μM Dorsomorphin. Control kinase assays were performed where the lysate (source of the kinase) was omitted. Kinase assay supernatants were probed by Western blot using anti-phospho ACC and anti-ACC antibodies. IP indicates antibody used for immunoprecipitation. WB indicates the antibody used for Western blotting. **(B)** The intensity of the anti-phospho ACC signal relative to the anti-ACC signal for each sample was determined. Each data point represents an independent kinase reaction. **P ≤ 0.01.

Previous studies have demonstrated that dorsomorphin inhibits AMPK in mammalian cells, leading to decreased AMPK α phosphorylation and inhibition of downstream processes regulated by AMPK (e.g., glycolysis) (Shen et al., 2008; Vucicevic et al., 2011; Liu et al., 2014). The paradoxical increase in glycolytic activity induced by dorsomorphin in schistosomes, together with the significant sequence differences that exist between schistosome AMPK α and the α subunits of other species, led us to question whether dorsomorphin was an effective inhibitor of schistosome AMPK. To address this question, we immunoprecipitated *S. mansoni* AMPK from adult schistosomes and assessed its kinase activity in the presence or absence of dorsomorphin, using human acetyl-coA carboxylase-1 (ACC) as substrate (Berg et al., 2002; Hardie et al., 2016). The extent of ACC phosphorylation was then assessed by Western blot using anti-ACC and anti-phospho-ACC antibodies (Figure 2A). Phosphorylation of ACC by immunoprecipitated schistosome AMPK was significantly reduced in the presence of 10 μM dorsomorphin (Figure 2B). These data suggest dorsomorphin is an effective inhibitor of *S. mansoni* AMPK and that the paradoxical effect of dorsomorphin on schistosome glycolytic activity is not explained by a failure of dorsomorphin to inhibit schistosome AMPK.

### Dorsomorphin Increases Phosphorylation and Expression of AMPK α in *S. mansoni* Adults

**FIGURE 3:**
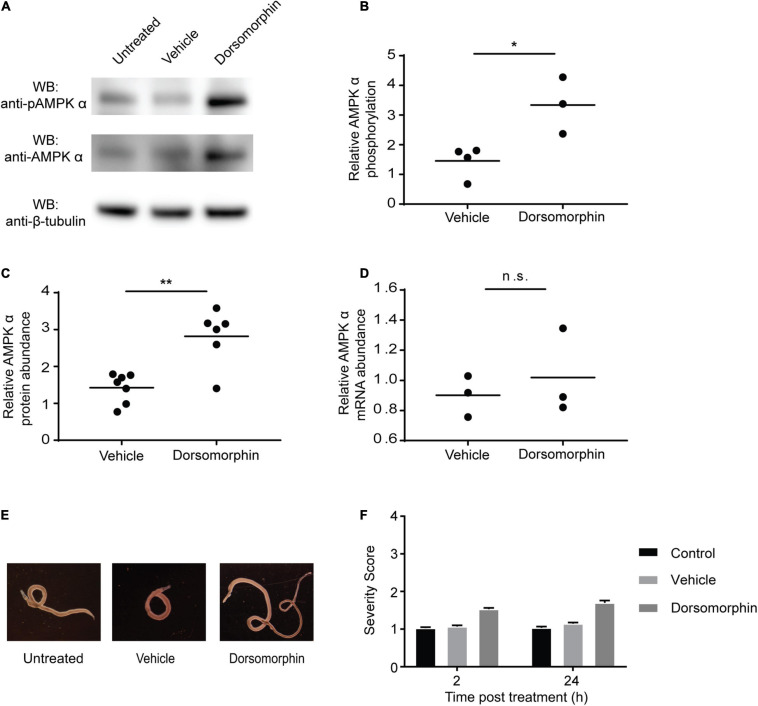
Chemical inhibition of AMPK induces compensatory increases in AMPK α protein and phosphorylation. **(A)** Freshly isolated adult schistosomes were incubated in either media alone (untreated) or media supplemented with 10 μM dorsomorphin or vehicle. After 24 h, lysates were prepared and equal amounts of protein were probed by Western blot using anti-pAMPK α antibody **(top panel)**, anti-AMPK α antibody **(center panel)**, and anti-β-tubulin antibody **(bottom panel)**. WB indicates the antibodies used for Western blotting. **(B)** Relative AMPK α phosphorylation under each condition was calculated by determining the ratio of anti-pAMPK α signal intensity to that of the anti-AMPK α signal intensity, and then normalizing to the intensity of the anti-β-tubulin signal to control for protein loading. Vehicle and dorsomorphin values shown are relative to untreated ratios (which were normalized to a value of 1). **(C)** Relative AMPK α protein abundance under each condition was calculated by determining the ratio of the intensity of the anti-AMPK α signal to that of anti-β-tubulin. Vehicle and dorsomorphin values shown are relative to untreated. **(D)** AMPK α transcripts of adult schistosomes exposed to the described conditions were quantified using quantitative real-time RT-PCR. AMPK α transcript abundance in vehicle and dorsomorphin samples relative to that of untreated samples are shown. Data points shown are biological replicates. **(E)** Freshly isolated adult schistosome pairs photographed after 24 h incubation in either medium alone (untreated) or medium supplemented with 10 μM dorsomorphin or vehicle. **(F)** Worms incubated in either medium alone (control) or medium supplemented with 10 μM dorsomorphin or vehicle were phenotypically scored after 2 and 24 h incubation in the aforementioned conditions. Data shown are averages of at least 3 biological replicates. ^ns^*P* > 0.05, *P ≤ 0.05, **P ≤ 0.01.

Previous studies have shown that dorsomorphin exposure inhibits mammalian AMPK phosphorylation and kinase activity (Liu et al., 2014; Cheng et al., 2019). However, when adult schistosomes were exposed to 10 μM dorsomorphin for 24 h, a significant increase in phosphorylation of AMPK α was detected by Western blot (Figures 3A, B). In addition, a significant increase in overall schistosome AMPK α protein abundance was also detected by Western blot in response to dorsomorphin treatment (Figure 3C). To determine if the increase in total AMPK α protein post-dorsomorphin exposure was due to an upregulation of AMPK α gene expression, we used quantitative RT-PCR to assess AMPK α mRNA abundance in adult schistosomes after 24 h of dorsomorphin exposure. No significant differences in AMPK α transcript content were found in untreated and dorsomorphin-treated schistosomes (Figure 3D). These data suggest that both post-transcriptional and post-translational mechanisms contribute to a compensatory increase in schistosome AMPK activity in response to dorsomorphin inhibition. Consistent with the finding that dorsomorphin failed to induce a loss of function of AMPK in adult schistosomes, we observed that incubation in 10 μM dorsomorphin for 24 h resulted in minimal phenotypic changes in cultured worms. Other than for an increased tendency for paired males and females to separate and detach from the bottom of the culture plate, no obvious phenotypic changes were induced by dorsomorphin exposure (Figure 3E). Quantification of phenotypic observations using a phenotypic scoring system (Kyere-Davies et al., 2018) further emphasized the absence of any significant phenotypic differences between dorsomorphin-treated and untreated or vehicle control-treated worms (Figure 3F).

### Inhibition of AMPK Is Toxic for Schistosomula

**FIGURE 4:**
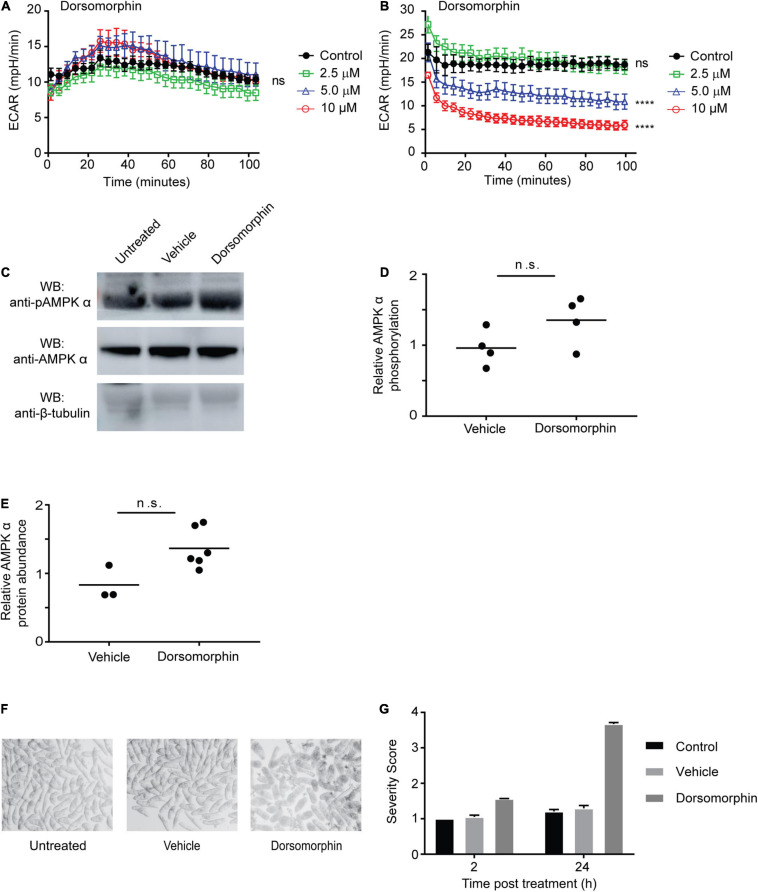
Chemical inhibition of AMPK is toxic for juvenile schistosomes. **(A,B)** The extracellular acidification rate (ECAR) of schistosomula (30 min post-transformation) immediately after exposure to dorsomorphin **(A)**, and after 48 h of exposure to dorsomorphin **(B)** was measured using the Agilent Seahorse XFe96. **(C)** Schistosomula (30 min post-transformation) were incubated in either medium alone (untreated) or media supplemented with 10 μM dorsomorphin or vehicle. After 24 h, lysates were prepared and equal amounts of protein were probed by Western blot using anti-pAMPK α antibody **(top panel)**, anti-AMPK α antibody **(center panel)**, and anti-β-tubulin antibody **(bottom panel)**. WB indicates the antibodies used for Western blotting. **(D)** Relative AMPK α phosphorylation under each condition was calculated by determining the ratio of anti-pAMPK α signal intensity to that of anti-AMPK α signal intensity, which was then normalized to the intensity of the anti-β-tubulin signal to control for differences in protein loading. Vehicle and dorsomorphin values shown are relative to untreated control. **(E)** Relative AMPK α protein abundance under each condition was calculated by determining the ratio of the intensity of the anti-AMPK α signal to that of anti-β-tubulin. Vehicle and dorsomorphin values shown are relative to untreated. Each data point shown represents a biological replicate. **(F)** Schistosomula (30 min post-transformation) were immediately placed in culture, in either medium alone (untreated) or medium supplemented with 10 μM dorsomorphin or vehicle, and photographed 24 h post-incubation. **(G)** Schistosomula incubated in either medium alone (control), or medium supplemented with 10 μM dorsomorphin or vehicle were phenotypically scored after 2 and 24 h incubation. Data shown are averages of at least 3 biological replicates. ^ns^*P* > 0.05, ****P ≤ 0.0001.

We previously showed that while cercariae and newly transformed schistosomula contain AMPK α protein, AMPK α transcripts are not detectable in these life cycle stages, suggesting the AMPK α gene is not actively transcribed at this stage of the life cycle (Hunter and Davies, 2018). Because adult schistosomes appear to avoid the toxic effects of dorsomorphin by upregulating AMPK α activity and protein abundance (Figure 3), we hypothesized that cercariae and schistosomula, which do not contain AMPK α transcript, might be more susceptible to dorsomorphin-induced toxicity. First, we evaluated whether AMPK inhibition induced any metabolic changes in schistosomula. Exposure of schistosomula to increasing concentrations of dorsomorphin did not significantly alter glycolytic activity when compared to control parasites (Figure 4A). This was in contrast to the dorsomorphin-induced increase in glycolytic activity we observed in adult worms (Figure 1). Furthermore, we found that prolonged exposure of schistosomula to dorsomorphin for 48 h resulted in significant dose-dependent loss of glycolytic activity (Figure 4B). When we examined the phosphorylation status and abundance of AMPK α protein in dorsomorphin-exposed schistosomula by immunoblot, we found no evidence of significant increases in AMPK α phosphorylation (Figures 4C, D) or overall protein levels (Figures 4C, E) in response to 10 μM dorsomorphin, consistent with our prediction that schistosomula would not be able to synthesize additional AMPK α due to the absence of AMPK α transcripts in this life cycle stage. Lastly, we noted that incubation of schistosomula in 10 μM dorsomorphin resulted in notable phenotypic changes compared to untreated and vehicle control-treated parasites. Schistosomula exposed to dorsomorphin appeared rounded, granular, and dark in appearance, while control schistosomula were translucent and maintained a rod-like shape (Figure 4F). Application of phenotypic scoring to dorsomorphin-exposed and control schistosomula produced largely similar scores after 2 h of exposure, but after 24 h of exposure, schistosomula treated with dorsomorphin exhibited severity scores approaching 4 (the highest severity score in the phenotypic scoring system; Figure 4G). This high score reflected the phenotypic changes that were evident in the dorsomorphin-exposed parasites, such as dark color, granular appearance and rounded shape (Figure 4F), as well as a loss of motility.

### Knockdown of *S. mansoni* AMPK α via RNAi Is Effective Yet Causes no Significant Impact on Adult Worm Fitness and Survival

**FIGURE 5:**
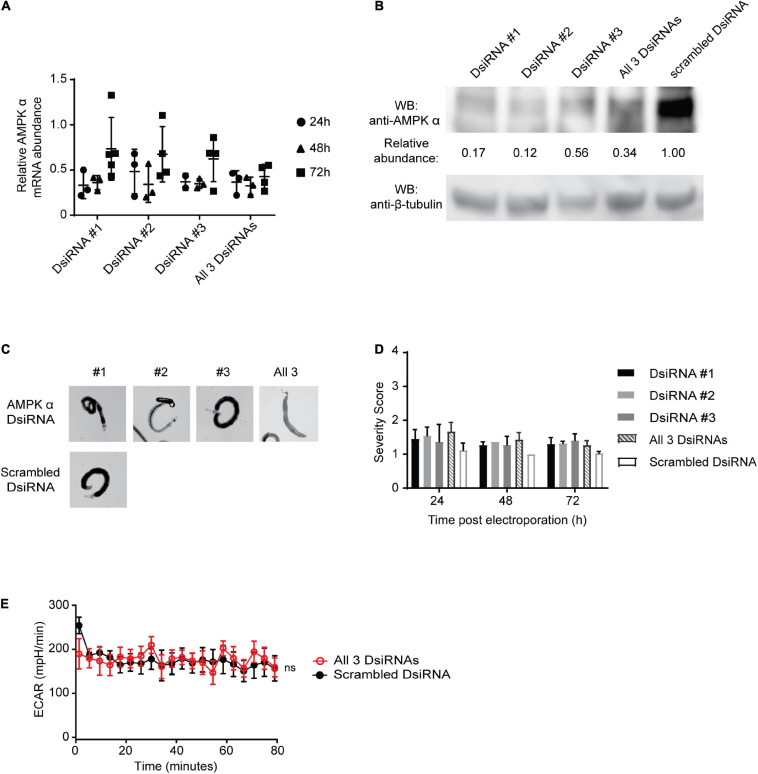
RNAi knockdown of AMPKα in adult schistosomes. **(A)** RNAi was performed on freshly isolated adult schistosomes by electroporation in the presence of three *S. mansoni* AMPK α-targeting DsiRNA constructs, either separately (DsiRNA #1, DsiRNA #2, and DsiRNA #3), or in combination (all three DsiRNAs). Control parasites were electroporated with a scrambled DsiRNA control construct. AMPK α transcripts were quantified at 24, 48, and 72 h post-RNAi using quantitative real-time RT-PCR. AMPK α transcript abundance relative to the scrambled DsiRNA control were calculated. Data points represent independent biological replicates composed of 5–7 worm pairs. Bars represent mean ± standard deviation. **(B)** Lysates of adult schistosomes 72 h post-RNAi were prepared and equal amounts were probed by Western blot using anti-AMPK α and anti-β-tubulin antibodies. Protein abundance was determined by normalizing the anti-AMPK α signal to the anti-β-tubulin signal for each sample. Relative protein abundance compared to the scrambled DsiRNA samples was then calculated. WB indicates the antibodies used for Western blotting. Western blot pictured is representative of at least 3 biological replicates. **(C)** Representative adult schistosomes electroporated in the presence of either AMPK α-targeting DsiRNA #1, #2, #3, all 3 constructs combined, or scrambled DsiRNA were photographed 72 h post-RNAi. **(D)** DsiRNA-treated adult worms were phenotypically scored at 24, 48, and 72 h post-electroporation. Data shown are averages of at least 3 biological replicates. **(E)** The extracellular acidification rate (ECAR) of adult *S. mansoni* pairs 72 h post-electroporation with either all 3 AMPK α-targeting DsiRNAs or scrambled DsiRNAs were measured using Agilent Seahorse XFe96 system. Each data point is the average of at least 3 biological replicates. Data are representative of at least two independent experiments. ^ns^*P* > 0.05.

To further assess the effects of reduced AMPK function in adult schistosomes, we attempted to reduce the abundance of AMPK α protein by RNAi-mediated targeting of the AMPK α transcript. We tested three different DsiRNAs designed to target the schistosome AMPK α mRNA at separate nucleotide positions. Adult schistosomes treated with 6 μM of either each DsiRNA alone or the combination of all three DsiRNAs exhibited at least 50% reductions in AMPK α transcripts within 24–48 h following electroporation (Figure 5A). By 72 h post-DsiRNA electroporation, AMPK α transcript levels rebounded in schistosomes that received each of the three DsiRNAs alone, returning to about 70% of transcript levels in control worms. Schistosomes that received the combination of all three DsiRNAs exhibited a more durable reduction in AMPK α transcripts, with mRNA abundance reduced to approximately 30% over the 72 h following electroporation. At 7 days post-DsiRNA electroporation, adult worms were collected to evaluate the effect of RNAi-mediated AMPK α transcript reduction on AMPK α protein abundance. All three DsiRNAs, individually and in combination, were successful in inducing a reduction in overall AMPK α protein, with 12–56% of protein remaining when compared to control worms (Figure 5B).

At 24, 48, and 72 h post-RNAi, adult worms were carefully examined for phenotypic changes (Figure 5C) and subjected to phenotypic scoring (Figure 5D). Modest phenotypic changes as a result of AMPK α knockdown were observed at 24 h post-DsiRNA electroporation, but these were not statistically significant, and the phenotypic effects of RNAi gradually became less apparent as the experiment proceeded. Phenotypic effects of AMPK α knockdown included incoordination, with worms temporarily losing their ability to remain suctioned to the tissue culture plate, decreased male/female pairing, and decreased parasite activity/motility. However, by 72 h post-DsiRNA treatment these effects had diminished and, phenotypically, worms appeared to return to their pre-RNAi state, as evidenced by the restoration of coordination/suctioning, and pairing, among other factors (Figure 5D). AMPK α targeting did not result in any apparent phenotypic changes at 7 days post-RNAi, when AMPK α-targeted worms were indistinguishable from control worms (data not shown).

Lastly, we tested whether reduced levels of AMPK α protein had any effect on adult worm glycolysis, by assessing worm glycolytic activity using the Seahorse platform at 72 h post-DsiRNA treatment. Consistent with the phenotypically normal appearance of the worms, we found that glycolytic activity of worms treated with the three AMPK α-specific DsiRNA and control scrambled DsiRNA were indistinguishable (Figure 5E), suggesting that, at least under our *in vitro* culture conditions, worm glycolytic activity is not diminished by AMPK α knockdown.

### Inhibition of Schistosome AMPK Leads to Dysregulation of Adult Schistosome Glycogen Stores

**FIGURE 6:**
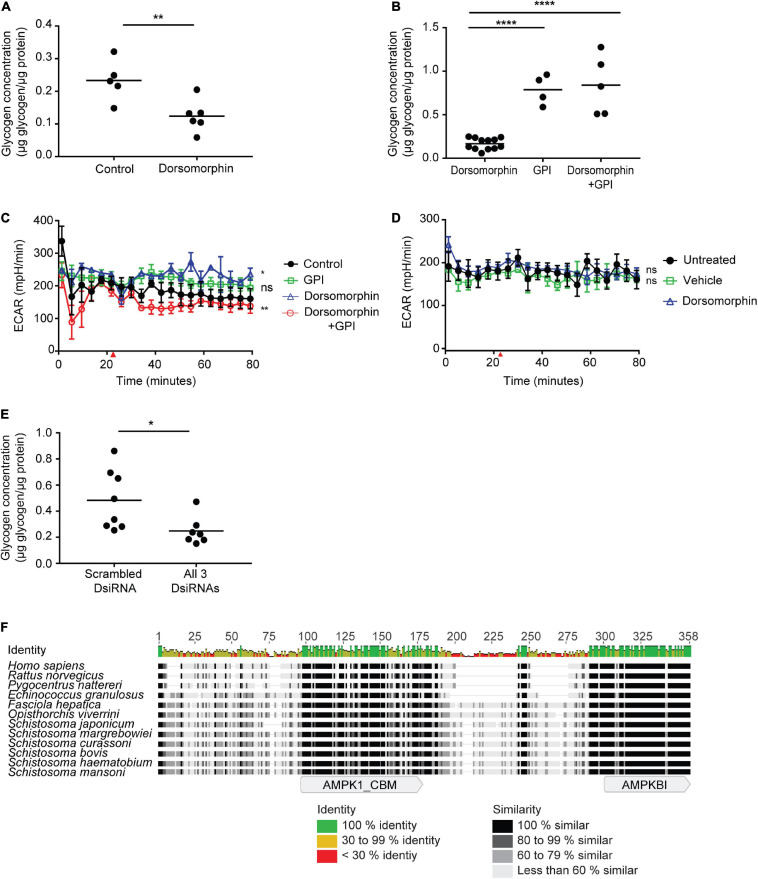
AMPK regulates glycogen metabolism in adult schistosomes. **(A)** Freshly isolated adult schistosomes were incubated in either medium alone (control) or medium supplemented with 10 μM dorsomorphin. After 24 h, lysates were prepared and glycogen content was measured. **(B)** Freshly isolated adult schistosomes were incubated in medium supplemented with 10 μM dorsomorphin, 75 nM glycogen phosphorylase inhibitor (GPI) or 10 μM dorsomorphin combined with 75 nM GPI. After 24 h, lysates were prepared and glycogen content was measured. In **(A,B)**, each data point indicates one schistosome pair, and graphs are representative of at least 3 independent experiments. **(C)** ECAR of adults worms exposed to 10 μM dorsomorphin, 75 nM GPI, or a both combined, was measured using the Agilent Seahorse XFe96 instrument. Red arrow indicates the time point at which compounds were introduced into parasite culture media. Each data point is the average of at least 3 biological replicates. **(D)** 72 h post-electroporation of adult *S. mansoni* worms with all 3 AMPK α-targeting DsiRNAs, ECAR upon exposure to 10 μM dorsomorphin was compared to vehicle, or no treatment, using the Agilent Seahorse XFe96 system. Red arrow indicates the time point at which compounds were introduced into parasite culture medium. Each data point is the average of at least 3 biological replicates. **(E)** Freshly isolated adult schistosomes were electroporated with either control scrambled DsiRNA or a combination of all 3 AMPK α-targeting DsiRNAs. 72 h post-electroporation, lysates were prepared and glycogen content was measured. Each data point represents a biological replicate. **(F)** The amino acid sequence of *S. mansoni* AMPK β (MN480434) aligned with AMPK β sequences of *Homo sapiens* (NP_006244), *Rattus norvegicus* (NP_114182.1), *Pygocentrus nattereri* (XP_017539403.1), *Echinococcus granulosus* (AER10555), *Fasciola hepatica* (THD24738.1), *Opisthorchis viverrini* (XP_009174364.1), *Schistosoma japonicum* (TNN15813.1), *Schistosoma margrebowiei* (VDP35519.1), *Schistosoma curassoni* (VDO62757.1), *Schistosoma bovis* (RTG86190.1), and *Schistosoma haematobium* (XP_012797764.1). The alignment shows sequence identity and similarity as well as the approximate locations of the glycogen binding domain (AMPK1_CBD) and C-terminal domain (AMPKB1) that associates with AMPK α and γ subunits. ^ns^*P* > 0.05, *P ≤ 0.05, **P ≤ 0.01, ****P ≤ 0.0001.

Because our data suggested that the glycolytic activity of adult worms is not dependent on AMPK, we explored other hypotheses regarding the function of AMPK in adult schistosomes. Given the considerable evidence that AMPK stimulates glycogen breakdown in mammalian cells by inhibiting glycogen synthase and activating glycogen phosphorylase (Hardie et al., 2012; Jeon, 2016), we hypothesized that schistosome AMPK might likewise function in regulating adult parasite glycogenolysis. Indeed, such a role for AMPK in adult schistosomes could explain the increase in glycolysis observed in adult schistosomes in response to dorsomorphin exposure (Figure 1), as increased glycogen breakdown stimulated by dorsomorphin-induced AMPK α activity could provide the fuel necessary for increased glycolysis (Figure 3). Consistent with this hypothesis, we found that adult schistosomes incubated in 10 μM dorsomorphin for 24 h contained significantly lower glycogen content than worms maintained in medium alone (Figure 6A). Next, we tested whether the dorsomorphin-induced depletion of worm glycogen content could be blocked by glycogen phosphorylase inhibitor (GPI) (Treadway et al., 2001; Somsák et al., 2003). Fresh adult worms were maintained *in vitro* in medium containing dorsomorphin, GPI, or a combination of both for 24 h. The decrease in glycogen content induced by dorsomorphin was blocked by GPI (Figure 6B). To test whether breakdown of glycogen was required for the dorsomorphin-induced increase in glycolytic activity, worms treated with dorsomorphin, GPI, or a combination of both were subjected to metabolic analysis using the Agilent Seahorse platform. The increase in schistosome glycolytic activity in response to dorsomorphin was abolished by GPI (Figure 6C), and indeed exposure to both dorsomorphin and GPI led to lower levels of glycolysis than in control worms. This result is consistent with the conclusion that the dorsomorphin-induced increase in adult worm glycolysis is fueled by glucose derived from the breakdown of parasite glycogen reserves. Next, we sought to test whether AMPK α is required for the dorsomorphin-induced increase in adult worm glycolysis. To accomplish this, worms were subjected to RNAi-mediated AMPK α knockdown first and then treated with dorsomorphin 72 h later. RNAi-mediated AMPK α knockdown abrogated the dorsomorphin-induced increase of adult worm glycolysis (Figure 6D), suggesting that AMPK α does mediate the effect of dorsomorphin on glycolysis. However, when we assessed the glycogen content of AMPK α-specific RNAi-treated worms and control parasites, we found that RNAi-mediated AMPK α knockdown by itself led to depletion of parasite glycogen reserves (Figure 6E). Therefore, the blockade of dorsomorphin’s induction of glycolysis by AMPK α-specific RNAi may be due instead to the depletion in these parasites of the glycogen needed to fuel an increase in glycolysis. In other organisms, chronic activation of AMPK has been found to indirectly increase glycogenesis, by increasing the amount of glycose-6-phosphate available for the synthesis of fresh glycogen (Jeon, 2016). AMPK therefore plays a role in the establishment and maintenance of cellular glycogen stores, in addition to regulating glycogen utilization (Hardie et al., 2012). Our finding that RNAi-mediated AMPK α knockdown led to depletion of parasite glycogen content suggests that schistosome AMPK may likewise play a role in maintaining parasite glycogen reserves.

Central to the regulation of cellular glycogen stores by AMPK is the ability of the AMPK complex to physically interact with glycogen, via a carbohydrate binding motif located within the AMPK β subunit. Because schistosome glycogen stores were depleted in parasites subjected to RNAi-mediated AMPK knockdown, we explored whether the *S. mansoni* AMPK β protein retains the glycogen binding domain found in the AMPK β subunits of other organisms. Searching the National Center for Biotechnology Information (NCBI) Nucleotide database identified a predicted mRNA sequence for the *S. mansoni* AMPK β subunit (accession number XM_018790591), which encodes for a putative AMPK β protein of 287 amino acids (accession number XP_018646108). The XM_018790591 RNA is predicted to be derived from genomic locus Smp_170160, located at position 223300 – 234844 on supercontig Sc0049 of the *S. mansoni* genomic sequence^1^. However, comparison of the putative XM_018790591 mRNA sequence with the proposed exon-intron structure of Smp_170160 revealed that 177 nucleotides from the 3′ end of exon 4 of Smp_170160 had been omitted from the XM_018790591 sequence. Thus the predicted mRNA derived from Smp_170160 would encode for a considerably larger protein of 346 amino acids. To address this discrepancy, we independently cloned and sequenced the AMPK β cDNA from adult *S. mansoni*. First, we used the XM_018790591 and Smp_170160-derived mRNA sequences to identify expressed sequence tags (ESTs) in the NCBI EST database that provided 5′ and 3′ untranslated sequence upstream and downstream of the putative open reading frame. Then primers designed to hybridize either side of the ORF were used to amplify the coding region in its entirety. The sequence of the resulting PCR product exactly matched the predicted exon sequences of Smp_170160, confirming the inclusion of the 177 nucleotides from exon 4 in the mRNA. This sequence is available in the NCBI Nucleotide database (accession number MN480434). Analysis of the predicted 346 amino acid sequence revealed that the *S. mansoni* AMPK β protein is considerably bigger than the β subunits of vertebrates (e.g., human, Norway rat, and fish), and the AMPK β of the cestode *Echinococcus granulosus* (Figure 6F). However, similarly sized AMPK β subunits have been predicted by the genome projects of several other trematodes, including five other species of schistosome, suggesting that an extended AMPK β subunit may be a conserved feature of trematode AMPKs, with the additional sequence concentrated in the C-terminal half of the polypeptide (Figure 6F). Despite these differences, considerable sequence identity and similarity exist between all the trematode sequences and those of vertebrates (e.g., 45% identity between the *S. mansoni* and human sequences), and analysis of the *S. mansoni* sequence using the NCBI Conserved Domain Database (CDD) readily identified an AMPK glycogen recognition site (AMPK1_CBM, accession number pfam16561) at amino acids 94–175 (*E*-value 4.37e-41), in addition to a characteristic AMPK β interaction domain (AMPKBI, accession number smart01010) at the C terminus (*E*-value 1.24e-25) (Figure 6F).

## Discussion

Because maintenance of blood glucose concentrations within narrow limits is essential for life of the vertebrate host, adult schistosomes likely never face situations similar to those encountered by free-living organisms, where the supply of energy substrates is limited. Hence, it could be argued that adult schistosomes have no requirement to control their consumption of glucose, and the prodigious rate by which schistosomes metabolize glucose by glycolysis is consistent with this argument (Bueding, 1950). We previously found constitutively high levels of AMPK α phosphorylation in freshly isolated adult schistosomes, indicating that schistosome AMPK is constitutively active under steady state conditions within the mammalian host. Indeed, we estimate schistosome AMPK in freshly isolated adult worms is approximately four times more active than human AMPK in HEK cells grown under glucose-replete *in vitro* conditions (Hunter and Davies, 2018). While comparisons between species and between whole organisms and cell lines should be interpreted with caution, these observations do suggest that, like glycolysis in intravascular life stages, schistosome AMPK activity is constitutively high, and perhaps close to maximal even under steady state conditions. Thus, while we previously demonstrated that phosphorylation of schistosome AMPK α could be modestly enhanced when parasites are exposed to AMPK agonists (Hunter and Davies, 2018), any downstream effects on energy metabolism could be difficult to detect if both AMPK activation and glycolysis are already occurring at high levels. Consistent with this model, neither of the AMPK agonists we tested, metformin and AICAR, caused any measurable increase in parasite glycolysis. There are other possible explanations for these observations. For example, while metformin and AICAR are well characterized AMPK agonists in other species, perhaps neither are especially potent in activating schistosome AMPK, and perhaps there are as yet undiscovered agonists of schistosome AMPK that would cause substantial increases in schistosome AMPK α phosphorylation and parasite glycolysis. Furthermore, we did not explore other potential effects of AMPK agonists on schistosomes, such as increased rates of autophagy. These possibilities will be explored in future studies.

Paradoxically, chemical inhibition of AMPK by dorsomorphin treatment resulted in an increase in adult schistosome glycolysis. This result was unexpected, as dorsomorphin treatment of live cells consistently reduces AMPK α phosphorylation and interrupts downstream metabolic processes (Vucicevic et al., 2011; Hu et al., 2017). These data led us to question whether dorsomorphin is actually capable of inhibiting schistosome AMPK, as there are significant differences in the sequence and size of *S. mansoni* AMPK α compared to those of other species (Hunter and Davies, 2018). However, results from an *in vitro* kinase assay confirmed that dorsomorphin does indeed inhibit the catalytic activity of the schistosome AMPK α subunit. Further analysis to understand the mechanism behind this unexpected increase in glycolysis revealed that dorsomorphin exposure also induced increases in AMPK α phosphorylation and overall AMPK α protein levels as well. We hypothesize that these increases represent a compensatory response by the parasite to surmount the inhibition of this critically important enzyme (Iskar et al., 2010). Though increased abundance of AMPK α protein post-dorsomorphin treatment could be detected via Western blot, no significant difference in transcript abundance was detected between untreated and dorsomorphin-treated adult schistosomes. However, transcript levels may not directly correlate with protein levels for several reasons (Liu et al., 2016; Becker et al., 2018). First, it is possible that a sudden loss of AMPK α activity due to dorsomorphin exposure could induce post-transcriptional regulatory mechanisms that result in a rapid increase in AMPK α protein synthesis from existing AMPK α mRNA (Day and Tuite, 1998; Torrent et al., 2018), which is abundant at this stage of the life cycle (Hunter and Davies, 2018). Second, studies in mammalian systems have identified microRNAs (miRNAs) that can directly target AMPK α mRNA and affect rates of protein synthesis (He et al., 2017). As there is already evidence that schistosomes utilize miRNAs for post-transcriptional regulation (Gomes et al., 2009; Hao L. et al., 2010; de Souza Gomes et al., 2011), it is possible that schistosome AMPK α protein abundance is also regulated by this mechanism. There are alternative explanations for the paradoxical increases in glycolysis and AMPK α phosphorylation induced by dorsomorphin in adult schistosomes. For example, it is possible that increases in AMPK α protein abundance and phosphorylation in response to dorsomorphin are unrelated to a direct effect of dorsomorphin on AMPK, as dorsomorphin is known to have activity against other signaling pathways, e.g., the bone morphogenetic protein (BMP) pathway (Yu et al., 2008; Hao J. et al., 2010; Vucicevic et al., 2011; Saito et al., 2012; Dasgupta and Seibel, 2018). While this pathway is conserved in schistosomes (Freitas et al., 2009), it is unclear how interruption of BMP signaling would result in increased glycolysis and AMPK α protein abundance. It is also possible that schistosome AMPK is indirectly activated by dorsomorphin. For example, some catalytic-site inhibitors of other kinases, like PKB/Akt (Okuzumi et al., 2009), exert an activating effect on AMPK by indirectly raising cellular AMP/ADP concentrations (Bain et al., 2007; Scott et al., 2014; Zadra et al., 2014), though why this would also result in an increase in AMPK α protein abundance is unclear.

In contrast to adult schistosomes, newly transformed schistosomula do not exhibit any increase in glycolytic activity in response to dorsomorphin, nor is there any detectable increase in AMPK α protein abundance or phosphorylation. This latter finding is predictable, as we had previously shown that AMPK α mRNA is virtually undetectable in schistosomula (Hunter and Davies, 2018), suggesting the gene is not actively transcribed in this developmental stage, and leaving the parasites without a means to compensate for the inhibition of AMPK α protein by dorsomorphin. Furthermore, newly transformed schistosomula exhibited significant phenotypic deterioration after exposure to dorsomorphin for 24 h, and diminished metabolic activity after 48 h, indicating parasite death. We therefore conclude that AMPK is essential to schistosomulum viability, and that an inability to synthesize *de novo* AMPK α protein underlies the sensitivity of schistosomula to dorsomorphin. While we cannot exclude the possibility that dorsomorphin mediates toxic effects against schistosomula via some other target, the correlation of the compensatory response of adult worms with their resistance to dorsomorphin, and the absence of this compensatory response in dorsomorphin-susceptible schistosomula, argues that a direct effect on dorsomorphin on schistosome AMPK α is the most parsimonious explanation for the anti-schistosomular effects of dorsomorphin.

The rapid upregulation of AMPK α protein abundance and phosphorylation by adult worms in response to dorsomorphin-mediated inhibition of AMPK suggests that AMPK fulfills an important role at this stage of the life cycle. We therefore sought to disrupt AMPK α expression by targeting AMPK α mRNA by RNAi. Using a combination of DsiRNAs, we were able to significantly deplete AMPK α mRNA and protein levels in adult schistosomes. However, loss of AMPK α protein did not result in any obvious phenotypic changes in the parasites, nor did it reduce steady-state schistosome glycolytic activity *in vitro*. These results suggest that schistosome glycolysis is not dependent on AMPK and proceeds regardless of AMPK expression and activation. We therefore explored other explanations for the rapid increase in glycolysis observed in dorsomorphin-treated adult schistosomes.

Interestingly, we noted that adult worm exposure to dorsomorphin resulted in depletion of parasite glycogen stores, consistent with the known role of AMPK in activating glycogen phosphorylase (Jeon, 2016). This finding suggested that glycogen breakdown might fuel the increase in parasite glycolytic activity induced by dorsomorphin exposure. Consistent with this hypothesis, treatment of adult worms with a glycogen phosphorylase inhibitor to prevent glycogen breakdown also blocked the dorsomorphin-induced increase in glycolysis. To establish whether dorsomorphin-induced glycogen depletion was mediated by AMPK, we attempted to test whether depletion of AMPK α protein by RNAi blocked dorsomorphin-induced glycolysis and glycogen utilization. However, we found that RNAi-mediated AMPK α depletion by itself led to significant reduction of parasite glycogen stores. This result unfortunately prevents us from drawing conclusions regarding the role of AMPK α in dorsomorphin-stimulated glycolysis, as an absence of a glycolytic response to dorsomorphin in these worms could simply be due to insufficient glycogen reserves. However, this result is perhaps to be expected – in other organisms, chronic AMPK activation has been found to indirectly promote glycogen synthesis and the maintenance of glycogen stores, by maintaining the intracellular glucose-6-phosphate concentrations that are required for glycogen synthesis (Hardie, 2011; Hardie et al., 2012; Janzen et al., 2018). The fact that AMPK is always readily detectable in an activated state in freshly isolated adult schistosomes (Hunter and Davies, 2018) suggests that AMPK is chronically activated in adult parasites and could therefore fulfill a similar role in maintenance of glycogen reserves. In an organism where glycolysis is the sole source of energy production, and where there is an unlimited supply of glucose to fuel high rates of glycolysis, perhaps there is little need for the regulation of glycolysis by AMPK, and this enzyme pathway has been conserved in schistosomes to fulfill other functions instead, such as maintenance of glycogen reserves. Indeed, resumption of AMPK expression after infection of the mammalian host (Hunter and Davies, 2018) may serve to re-establish glycogen stores now that the parasite has penetrated a glucose-replete environment, as little glycogen is transported from molluscan host to mammalian host by the cercaria (Horemans et al., 1992; Skelly et al., 1993) and schistosomula do not contain detectable glycogen (data not shown). Conservation of the carbohydrate binding motif in the *S. mansoni* AMPK β subunit is consistent with these hypotheses (McBride and Hardie, 2009; Herzig and Shaw, 2018).

Given the stability and consistency of blood glucose concentrations maintained by the vertebrate host, it is unclear what the function of adult schistosome glycogen stores might be. Previous work has shown that adult schistosome glycogen stores are highly dynamic, undergoing constant breakdown and re-synthesis (Tielens et al., 1990a,b). It has been speculated that perhaps adult schistosome pairs switch to glycogen as a glucose source during oviposition, when the parasites squeeze into narrow venules where blood flow might be reduced or occluded altogether for brief periods. However, reports from other organisms suggest glycogen may fulfill roles beyond serving as merely a backup energy source. For example, in mammalian cells there is evidence that glycogen contributes to protection against oxidative stress, due to its preferential use as a substrate for the pentose phosphate pathway that produces NADPH, which neutralizes reactive oxygen species (Singh et al., 2007; Favaro et al., 2012; Ros and Schulze, 2012). Similar observations regarding the connection between glycogen and oxidative stress have been noted in the free-living nematode *Caenorhabditis elegans* (Gusarov et al., 2017). As schistosomes are presumably subject to constant oxidative pressure from the host’s immune system (Sayed et al., 2006; Mourao Mde et al., 2009), these proposed alternative functions of glycogen could be important. If a primary function of AMPK in adult schistosomes is to regulate the turnover of glycogen stores, targeting AMPK *in vitro* might not result in any obvious detrimental effects due to the absence of significant oxidative stress. However, targeting AMPK *in vivo* might render the parasites more susceptible to oxidative damage.

## Conclusion

Our data suggest that schistosome AMPK fulfills different roles in larval and adult parasites. In the schistosomulum, when the parasite is transitioning to life inside the definitive host, AMPK α is required for viability, and as the AMPK α gene is not actively transcribed at this point, schistosomula might be especially vulnerable to AMPK targeting. These observations suggest that AMPK α may be a useful target for interventions aimed at preventing the establishment of a schistosome infection. In adult worms, our initial hypothesis that AMPK regulates the production of energy by glycolysis is not supported by our data, which instead suggest that glycolysis and AMPK activation are uncoupled in this developmental stage. However, AMPK appears to still play an important role in adults, as AMPK inhibition induces a compensatory upregulation in AMPK α abundance and phosphorylation. While this compensatory mechanism makes it difficult to test the function of AMPK α in adult parasites, our data point to a significant role in the regulation of parasite glycogen stores, the function of which has been the subject of much speculation. Our data provide fresh insights into the regulation of energy substrate utilization in a parasite that capitalizes on host glucose homeostasis and its anatomic location in the host bloodstream, and suggest that parasite energy metabolism may yet exhibit unique features that can be targeted to the detriment of the parasite.

## Data Availability Statement

The datasets generated for this study can be found in online repositories. The names of the repository/repositories and accession number(s) can be found below: https://www.ncbi.nlm.nih.gov/genbank/, MN480434.

## Ethics Statement

The animal study was reviewed and approved by Institutional Animal Care and Use Committee (IACUC) of Uniformed Services University (permit number A3448-01).

## Author Contributions

KH, AM, MM-K, and SD contributed to conception and design of the study. KH and AM performed the experimental work. KH analyzed the data and wrote the first draft of the manuscript. All authors contributed to manuscript revision, read, and approved the submitted version.

## Author Disclaimer

The opinions and assertions expressed herein are those of the author(s) and do not necessarily reflect the official policy or position of the Uniformed Services University or the Department of Defense.

## Conflict of Interest

The authors declare that the research was conducted in the absence of any commercial or financial relationships that could be construed as a potential conflict of interest.

## Publisher’s Note

All claims expressed in this article are solely those of the authors and do not necessarily represent those of their affiliated organizations, or those of the publisher, the editors and the reviewers. Any product that may be evaluated in this article, or claim that may be made by its manufacturer, is not guaranteed or endorsed by the publisher.

## Footnotes

1
www.genedb.org/#/chromosome/Schisto_mansoni.SC_0049
